# Editorial: Emerging talents in computational genomics

**DOI:** 10.3389/fgene.2024.1512594

**Published:** 2024-11-05

**Authors:** Junichi Iwata, Kenta Nakai

**Affiliations:** ^1^ Department of Orthodontics and Pediatric Dentistry, University of Michigan School of Dentistry, Ann Arbor, MI, United States; ^2^ Department of Diagnostic and Biomedical Sciences, The University of Texas Health Science Center at Houston, Houston, TX, United States; ^3^ The Institute of Medical Science, The University of Tokyo, Tokyo, Japan

**Keywords:** computational genomics, gene-based prediction model, host-pathogen interaction genetics, transcriptome data analyses, metagenomics

The aim of this Research Topic, “*Emerging Talents in Computational Genomics*” at *Frontiers in Genetics*, was to highlight the emerging talents of student researchers in computational genomics and give them opportunities to get involved in journal editorial processes. Behind this initiative, the journal was aware that results from most student-driven research projects are not much shared with a broader audience, while many students are undertaking research in computational genomics as part of their educational programs. Therefore, we invited student researchers to this Research Topic at the Frontiers, providing them with hands-on guidance and constructive feedback.

As briefly summarized below, the authors successfully shared scientific findings from their research projects.

Mendapara introduced a novel gene-based prediction model for detecting chronic kidney disease, utilizing gene expression profiling datasets from the Gene Expression Omnibus (GEO) database. The model, constructed and optimized using a training dataset, was further validated with another dataset. This study identified genetic biomarkers for chronic kidney disease risk, which makes a significant contribution to the field.

Kabir et al. delved into the interactions between human and *Candida albicans* proteins in oral candidiasis, using datasets from the Human Protein-*C. albicans* Interaction Database. The authors identified corresponding human proteins by mapping differentially expressed genes in patients with *C. albicans*. This study not only provided a molecular map of the host-pathogen interaction in oral candidiasis but also potential targets for therapeutic intervention.

Rockenbach et al. analyzed transcriptome data from the human ovary and testis during the prenatal period and adulthood to study the role of gametes in sex-specific events such as oogenesis and spermatogenesis. The authors found that meiosis-related genes were differentially expressed in female and male gonads and gametes between normal and pathological conditions. These findings shed light on new candidate genes involved in human fertility disorders.

Chaudhari et al. reviewed the history of the development of microbe culture-independent molecular methods of metagenomics and reviewed techniques, data analyses, and data interpretation and representation.

Overall, the original research and review articles presented in this Research Topic underscore the importance of student-driven studies in computational genomics. As Research Topic Editors, we extend our heartfelt thanks to all the contributors for their invaluable peer-review processes and collaborative efforts, which we deeply appreciate. We believe this Research Topic will help identify talented students and allow the community to follow the promising careers of these emerging, talented researchers.

